# Emotional competence, attachment, and parenting styles in children and parents

**DOI:** 10.1186/s41155-022-00208-0

**Published:** 2022-03-14

**Authors:** Zeinab Mortazavizadeh, Lars Göllner, Simon Forstmeier

**Affiliations:** Developmental Psychology and Clinical Psychology of the Lifespan, Department of Psychology, University of Siegen, Adolf-Reichwein-Str. 2a, 57068 Siegen, Germany

**Keywords:** Emotional competence, Attachment, Parenting style, Children-parents

## Abstract

The goal of this study was to examine whether a subject’s emotional competence correlates to attachment styles and parenting styles in children and their parents. The study was conducted with fifty children (9–11 years old) and their parents, both of whose emotional competence (EKF) and parenting style (PAQ) were measured. The attachment styles of parents and children were measured with the Adult Attachment Scale (AAS) and the Bochumer Bindungstest (BoBiTe), respectively. The findings provide initial support to the assumption that attachment is related to emotional competence in parents. This relationship, however, was not significantly correlated in children. In addition, authoritative parenting and permissive parenting were significantly associated with emotional competence in parents. Emotional competence in children showed to be associated with an authoritative parenting style.

The significance of emotional competence in psychological stages of life has been demonstrated in various studies. Emotional competence is defined by Saarni (1990) as “the demonstration of self-efficacy in the context of emotion-eliciting social transactions” (p. 116). To expand on this definition, emotional competence can also be seen as the ability to understand and regulate the expression of emotions individually and in response to others, which can be used to ease cognitive tasks like problem solving (Gross, 2007; Depape et al., 2006). A greater emotional competence is associated with better well-being, life satisfaction and higher self-esteem (Schutte et al., 2002; Gallagher & Vella-Brodrick, 2008) as well as fewer psychological disorders (Mikolajczak et al., 2007). Moreover, research indicated that emotional competence is associated with better social and marital relationships (Schutte et al., 2001; Lopes et al., 2004, 2005). Developing children’s emotional competence is a basic necessity for academic and social success (Denham, 2006). A robust body of evidence has shown the significant role of parents’ responses to children’s emotions in promoting or inhibiting the development of emotion regulation in children (Denham et al., 1997, 2007; Eisenberg et al., 1996; Thompson & Meyer, 2007). Furthermore, it has been shown that there is a relationship between the expression of emotions in parents and socioemotional competence in children (Eisenberg et al., 1998).

## Emotional competence and attachment style

Decades of research have solidified support for the hypothesis that attachment is related to expression and regulation of emotion (Mikulincer & Shaver, 2007; Cassidy, 1994; Thompson, 1999). According to the National Scientific Council on the Developing Child (2004, 2007a, b), expressing emotions in early childhood is the way children start to communicate, and children with secure attachment possess the ability such as coping or problem solving through emotion regulation (Psouni & Apetroaia, 2014; Contreras et al., 2000; Abraham & Kerns, 2013; Kerns et al., 2007). Schore (2003) found that the co-regulation through the infant–parent relationship impacts the emotional development of children and that parents thereby help to enhance the child’s abilities to express and modulate their thoughts and emotional responses.

Attachment theory postulates that children’s emotional performance is affected in all phases by child-caregiver relationships (De Rosnay & Harris, 2002; Cassidy & Shaver, 2018). Children acquire the ability to express their emotions through observing the attachment figures (Denham et al., 2010) and emotion regulation develops in the context of relationships with caregivers (Gross, 2007). Furthermore, it has been proposed that children with secure attachment who have interacted with sensitive and flexible parents tend to express their emotions properly and learn how to manage their negative emotions in coping with stressful situations (Contreras & Kerns, 2000). Early attachment theory emphasized that emotions, behavioral strategies, and affect regulation in relationships are impacted by adult attachment (Shaver & Mikulincer, 2002; Gillath & Shaver, 2007; Mikulincer & Shaver, 2007).

The literature has shown that there is a significant correlation between patterns of maternal attachment and emotional competence in children (Stefanovic-Stanojevic et al., 2015). This is exemplified by the fact that unlike insecure mothers, mothers with a secure attachment were more supportive and aided their children in coping with problems (Crowell & Feldman, 1988). This is also supported by the study of Panfile and Laible (2012), who have demonstrated that children with secure attachment achieved a higher degree of emotion regulation, indicating that attachment style and emotion regulation are linked. Furthermore, results suggested that children with insecure attachment experience more difficulties with emotional competence (Vondra et al., 2001; Kidwell et al., 2010; Fagot & Pears, 1996; DeVito & Hopkins, 2001). By contrast, children with a secure attachment style showed a superior social, academic, and emotional function compared to their peers (Weinfeld et al., 2008). The positive correlation of secure attachment and social competence was also corroborated by Kerns et al. (1996) and evidence indicates that, unlike insecure parents, secure parents have more positive interactions with their children (Adam et al., 2004; Crowell & Feldman, 1988; Pearson et al., 1994). The study conducted by Kerns et al. (2007) also found a positive association between attachment relationships and better regulation of emotion, which supports the notion that emotion regulation development and attachment belong together.

## Emotional competence and parenting styles

The link between parenting style and child’s development has been demonstrated time and again throughout decades of research (Bowlby, 1969; Ainsworth et al., 1978) and that the parenting style has a primary role in children’s social and emotional development (Froiland et al., 2013; Aunola et al., 2015; Hart et al., 2003; Goodnow & Collins, 1990; Bornstein, 2006), while other studies have also shown that there is an association between parental emotions and parenting behavior (Dix, 1991; Lovejoy et al., 2000). Gottman et al. (1997), for example, demonstrated that there is an association between poor emotion regulation in children and parenting style. It has been argued that a dismissive parenting style is associated with inadequate emotion regulation (Ramsden & Hubbard, 2002) and that, conversely, positive parenting boosts children’s social competence (Brody et al., 2002), which highlights that there might be a correlation between emotional competence and parenting style. Furthermore, results revealed that parenting behavior is assumed to be a mediator in the relation between children's temperament and their problem behavior (Rothbart & Bates, 1998).

The findings indicate that children whose interactions with their parents were characterized by warmth and positive emotions show high levels of social competence and a diminution of hostility and internalizing problems (Scaramella et al., 1999; Matthews et al., 1996; Rothbaum & Weisz, 1994), adding to the evidence that there is a link between emotional competence in children and emotional competence in parents.

## Goal of this study

Despite growing understanding of the development of emotional competence, the awareness for its pivotal role in children and given its importance in human development, there is still a great need to examine its dimensions and its relation to factors such as attachment style and parenting style in parents and children. To the best of our knowledge, little attention has been paid to the interrelations among these sets of variables. Thus, the current study sought to investigate whether attachment style and parenting style are associated with emotional competence. Moreover, few empirical studies have been done on child–father–mother relationship with regard to their emotional competence, attachment styles, and parenting style variables. Therefore, the second aim of this study is to provide and extend existing research on parent–child dyads, considering the above-mentioned variables in the child–father–mother triad. Furthermore, to our knowledge, few studies have covered different aspects of emotional competence in children (9–11 years old) and their parents. The goal of this study was to look into examining the emotional competence of children and their parents, and this issue has expanded in line with previous research.

## Method

### Participants

The sample of respondents comprised families (*N* = 50) including fathers, aged 33–59 (*M* = 45.3 years), mothers, aged 32–53 years (*M* = 42.1 years), and their children aged between 9 and 11 years old (*M* = 10.4 years, 51.9% girls). The majority of children who took part in the study were in secondary school (68.5%) and 31.5% of them were in primary school. Only 4.7% of parents lived alone and most children lived in a family with both parents. Also, only families with very good skill in German were considered for participation in this study. As a consequence, all children and most parents (mothers 94.4%, fathers 94.4%) were born in Germany and their German language level specified as native. 58.6% of parents were educated and holding a BA or MA university degree. The rest of the parents had occupational training (27.8%), other qualification (13%), and 1.8% of parents had no educational degree or occupational training. Participants were recruited using advertisement flyers in different primary and secondary schools and in public places such as sport and dance clubs, hospitals, and toy stores in Siegen and Marburg and the nearby areas.

### Measures

#### Emotional competence

Emotional competence was assessed using the Emotional Competence Questionnaire (Emotionale-Kompetenz-Fragebogen, EKF; Rindermann, 2009). It is based on self-reports or reports by others and consists of 20 items on a five-point Likert-type scale ranging from 1 (strongly disagree) to 5 (strongly agree) which evaluate the following aspects of emotional competence: emotional expression, emotion regulation, regulation of the emotion of others, recognition of own emotion, and recognition of the emotion of others. The EKF has been found to have an excellent internal consistency (Cronbach’s *α* = 0.88–0.92). The EKF has been reported to be significantly related to more problems in players addicted to massively multiplayer online role-playing games and regulating their own emotions as well as understanding the emotional states of others (Leménager et al., 2013), providing evidence of its external validity.

#### Attachment style in children and parents

Attachment dimensions in parents were assessed with the German version of the Adult Attachment Scale (AAS, Collins & Read, 1990; German version: Schmidt et al., 2004). The AAS is a self-report measure which evaluates three dimensions of attachment, i.e., to what extent the subject is able to rely others (“depend”), to what extent the subject is afraid to lose their partner (“anxiety”), and to what extent the subject feels close to their partner (“close”). Secure attachment is characterized by high scores on the close and depend subscales and a low score on anxiety subscale. The reliability of the scale was verified and displayed quiet a good result (*α* = 0.72–0.79). Higher scores on the “Anxiety” subscale and lower scores on the “Depend” subscales of the AAS (Collins & Read, 1990; German version: Schmidt et al., 2004) were found to be associated with social anxiety in a study by Notzon et al. (2016).

Attachment styles in children were measured with the Bochumer Bindungstest (BoBiTe; Trudewind & Steckel, 2009), which is designed to evaluate attachment styles in children aged from 8 to 13 and using a semi-projective method which evaluates three primary types of attachment: secure, avoidant, and ambivalent (as identified by Ainsworth & Bell, 1970). The assessment comprises eleven pictures with every single picture covering a maximum of four different complexes. Each complex consists of three statements and during the test participants are asked to choose one statement out of three regarding the picture which describes their attachment-related situation. Choosing every item of a single complex leads to one of three attachment types (secure, insecure-ambivalent or insecure-avoidant). The total score indicates the final outcome and thus the child’s attachment style. The Cronbach’s *α* values were 0.82 for the secure scale, 0.70 for the insecure-avoidant scale, and 0.68 for the insecure-ambivalent scale (Trudewind & Steckel, 1999; Höner, 2009).

#### Parenting style in children and parents

The parenting style was assessed by using the German version of the Parental Authority Questionnaire (PAQ; Buri, 1991; Castello & Hubmann, 2014). The PAQ contains 30 items which are divided into 10 statements for each of the three types of parenting styles, including authoritative (10 items), authoritarian (10 items), and permissive (10 items). Children and parents were asked to complete the questionnaire on a 5-point Likert-type scale (“1 = strongly disagree, 2 = disagree, 3 = undecided, 4 = agree, or 5 = strongly agree”). Internal consistency of the German version was determined to be good to satisfactory on all scales (Cronbach’s alpha: 0.77–0.84), and the questionnaire showed good­retest reliability (*r* = 0.78–0.90) (Castello & Hubmann, 2014).

### Procedure

The study was carried out in accordance with the ethical standards and The Ethical Board of the German Psychological Society (DGPs), which authorized the study in advance (protocol number: LG 082016). Data were collected between January 2017 and July 2018. Before filling out the questionnaires, participants were given complete explanations of the aims and procedures of the study in oral and written form, and they completed and signed the informed consent form upon arrival at the laboratory. Two researchers conducted the face-to-face assessment in such a way that one of them assessed the child in one room, the other assessed the father and mother in another room, and instructions and explanations were given together. Therefore, the parents and one experimenter stayed in the room while the child and a second experimenter went to a nearby room. The participants began filling out the questionnaires at this time completed a set of questionnaires measuring different dimensions including attachment style, parenting style, and emotional competence, which was completed only by the parents. In the rare case the children had difficulties to understand an item, it was reformulated by the researcher. The duration of assessment was about 1 h in the case of the child and 1.5 h in the case of the parents. Each family received a monetary compensation of 50 euros and also a cinema voucher for their participation in this study.

### Statistical analysis

In this correlational study, descriptive statistics and correlation for all variables were conducted to estimate the relationship between attachment style, emotional competence, and parenting style in children and their parents.

## Results

### Children’s and father-mother-parents’ emotional competence

To examine the associations between the emotional competence of children and that of their parents, correlation coefficients were computed. The results show that there are positive associations between children’s and parents’ emotional competence in some, but not all facets (see Table 1). Children’s recognition of own emotions did not correlate with parental recognition of own emotions. Children’s recognition of the emotions of others correlated only with the mothers’ recognition of the emotions of others (*r* = .35, *p* < .05), but not with the fathers’ (*r* = − .02, n.s.). Children’s emotion regulation did not correlate significantly with parental emotion regulation. Children’s emotional expression correlated with the fathers’ emotional expression (*r* = .31, *p* < .05), but not the mothers’ (*r* = − .07, n.s.). And children’s regulation of the emotions of others correlated with the fathers’ regulation of the emotions of others (*r* = .30, *p* < .05), but not with the mothers’ (*r* = .18, n.s.). Children’s regulation of the emotions of others correlated with parent’s regulation of the emotions of others (parents: average of the mother and father values) (*r* = .30, *p* < 0.05). Also, parent’s recognition of emotion of others was significantly associated with children’s emotional expression and regulation of the emotion of others respectively (*r* = .36, *p* < 0.05; *r* = .53, *p* < 0.01). In addition, there are several significant intercorrelations between the emotional competences’ scales.

**Table 1:** Correlation between children’s and father–mother–parents’ emotional competence

	Emotional competence	Recognition of own emotions (children)	Recognition of the emotions of others (children)	Emotion regulation (children)	Emotional expression (children)	Regulation of the emotions of others (children)
Mother	Recognition of own emotions	.05	.13	.08	.18	.12
	Recognition of the emotions of others	.13	.345*	.23	.17	.328*
	Emotion regulation	.00	− .07	.12	.13	.00
	Emotional expression	− .16	.28	.16	− .07	.302*
	Regulation of the emotions of others	.07	.29	.06	.14	.18
Father	Recognition of own emotions	.01	.07	.08	.22	.06
	Recognition of the emotions of others	− .07	− .02	− .08	.328*	.417**
	Emotion regulation	.17	− .1	.05	− .03	.11
	Emotional expression	.09	.03	.05	.306*	.19
	Regulation of the emotions of others	.27	.22	− .21	.297*	.298*
Parents	Recognition of own emotions	.04	.17	.11	.293*	.13
	Recognition of the emotions of others	.05	.2	.09	.367*	.529**
	Emotion regulation	.13	− .11	.13	.06	.08
	Emotional expression	− .04	.2	.14	.17	.347*
	Regulation of the emotions of others	.21	.336*	− .08	.27	.301*

*p < 0.05, **p < 0.01

### Children and parents’ emotional competence and attachment styles

There are several significant associations between parents’ emotional competence and attachment style (see Table 2). Emotional expression in mothers was positively correlated with the close (*r* = .37, *p* < .01) and depend (*r* = .35, *p* < .05) subscales of attachment style. Also, regulation of emotion of others was significantly correlated with attachment depend in mothers (*r* = .43, *p < 0.01*). In fathers, emotional expression showed a correlation of *r* = .30, *p* < .05 and *r* = .45, *p* < 0.01 with attachment close and attachment depend respectively. Also, recognition of the emotions of others in fathers, but not in mothers, was positively associated with the close (*r* = .30, *p* < .05) and depend (*r* = .39, *p* < 0.01) subscales and negatively with the anxiety subscale (*r* = -.29, *p* < .05). Attachment depend in fathers also showed a positive association with recognition of emotions (*r* = .035, *p* = *p < 0.01*). There were several other significant, but also non-significant correlations (see Tables 2, 3, 4, and 5).

**Table 2:** Correlation between parents’ emotional competence and attachment styles

	Attachment styles	Recognition of own emotions	Recognition of the emotions of others	Emotion regulation	Emotional expression	Regulation of the emotions of others
Mother	Attachment close	.24	.17	.17	.372**	.07
	Attachment depend	.19	.2	− .01	.346*	.422**
	Attachment anxiety	.03	− .18	.02	.1	− .02
Father	Attachment close	.22	.300*	.316*	.296*	− .01
	Attachment depend	.357**	.386**	.08	.449**	.18
	Attachment anxiety	− .001	− .290*	.03	− .002	− .03
Child	Secure attachment style	.08	.19	.06	.08	− .03
	Insecure avoidant attachment style	.07	− .19	− .04	− .01	.03
	Insecure ambivalent attachment style	− .14	− .13	− .04	− .16	− .1

*p < 0.05, **p < 0.01

**Table 3:** Correlation between children’s emotional competence and attachment styles

	Attachment styles	Recognition of own emotions	Recognition of the emotions of others	Emotion regulation	Emotional expression	Regulation of the emotions of others
Mother	Attachment close	− .17	.09	.23	− .08	.22
	Attachment depend	− .18	.19	.09	.06	.21
	Attachment anxiety	.15	− .28	− .02	− .05	− .316*
Father	Attachment close	.06	.1	.24	.04	.23
	Attachment depend	− .03	.25	.12	.14	.339*
	Attachment anxiety	− .1	− .11	.05	− .11	− .14
Child	Secure attachment style	− .21	.1	− .06	− .01	.07
	Insecure avoidant attachment style	.26	.02	.24	− .1	− .1
	Insecure ambivalent attachment style	.04	− .19	− .15	.05	− .004

*p < 0.05, **p < 0.01

**Table 4:** Correlation between mothers’ emotional competence and attachment

	Attachment styles	Recognition of own emotions	Recognition of the emotions of others	Emotion regulation	Emotional expression	Regulation of the emotions of others
Mother	Attachment close	.27	.11	.19	.448**	.16
	Attachment depend	.22	.04	.11	.475**	.453**
	Attachment anxiety	− .11	− .06	− .11	.06	− .06
Father	Attachment close	.11	.289*	.13	.12	− .12
	Attachment depend	.18	.19	.1	.367**	− .03
	Attachment anxiety	.01	− .290*	− .05	.08	.04
Child	Secure attachment style	.03	.07	.282*	.08	.07
	Insecure avoidant attachment style	.18	.1	− .07	.04	.14
	Insecure ambivalent attachment style	− .27	− .25	− .358**	− .22	− .326*

*p < 0.05, **p < 0.01

**Table 5:** Correlation between fathers’ emotional competence and attachment

	Attachment styles	Recognition of own emotions	Recognition of the emotions of others	Emotion regulation	Emotional expression	Regulation of the emotions of others
Mother	Attachment close	.03	.13	.08	.1	− .07
	Attachment depend	.01	.23	− .07	.01	.17
	Attachment anxiety	.17	− .16	.09	.11	.03
Father	Attachment close	.2	.17	.279*	.313*	.13
	Attachment depend	.312*	.360**	.03	.285*	.340*
	Attachment anxiety	− .01	− .14	.05	− .07	− .1
Child	Secure attachment style	.1	.18	− .09	.03	− .13
	Insecure avoidant attachment style	− .12	− .337*	− .02	− .06	− .11
	Insecure ambivalent attachment style	.12	.05	.16	.01	.23

*p < 0.05, **p < 0.01

There was no significant correlation between parents’ emotional competence and children’s attachment style. Children’s emotional competence (regulation of the emotion of others) was significantly correlated with attachment anxiety in mothers and attachment dependence in fathers respectively (*r* = − .31 *p* < .05; *r* = .33, *p* < .05). Moreover, emotion regulation in mothers was positively associated with secure attachment style (*r* = .28, *p* < .05) and negatively associated with insecure ambivalent attachment style in children (*r* = − .35, *p* < 0.01) (see Table 4). Insecure avoidant attachment in children was also negatively associated with recognition of the emotions of others in fathers (*r* = − .33, *p* < .05) (see Table 5). Fathers and mothers’ emotional competence showed significant correlation with attachment. For instance, emotional expression in mothers was significantly associated with attachment close and attachment depend respectively (*r* = .45, *p* < 0.01; *r* = .47, *p* < 0.01) (see Table 4). In fathers, regulation of the emotion of others, emotional expression, and recognition of the emotion of others were associated with attachment depend respectively (*r* = .34, *p <* 0.05; *r* = .28, *p* < 0.05; *r* = .36, *p* < 0.01) (see Table 5).

### Children’s and parents’ emotional competence and parenting style

Correlation analyses were then conducted between the emotional competence (parents-children) and parenting style. The results show significant correlations between authoritative parenting in children and parents and regulation/recognition of the emotions of others in the parents’ subscales. Authoritative parenting (children’s view) was significantly correlated with recognition of emotion of others in mothers (*r* = .31, *p* < .05) and in fathers (*r* = .43, *p < .01*). In fathers, also, emotion regulation and regulation of the emotions of others were positively correlated with authoritative parenting (children’s view) respectively (*r* = .35, *p* < .05; *r* = .39, *p* < .05). In addition, permissive parenting in parents (average of the mother and father values and mothers represented a negative significant association with emotion regulation in parents, respectively (*r* = − .29, *p* < .05; *r* = -.33, *p* < .05) (see Table 6).

**Table 6:** Correlation between parents’ emotional competence and parenting styles

Parenting styles	Recognition of own emotions	Recognition of the emotions of others	Emotion regulation	Emotional expression	Regulation of the emotions of others
**Authoritative**					
*Children’s view*					
Mother	.29	.315*	.16	.15	.25
Father	.21	.431**	.349*	.11	.394*
Parents	.29	.409*	.3	.14	.357*
*Parents’ view*					
Parents	.08	.26	.25	− .01	.05
Mother	.2	.288*	.27	.1	.14
Father	− .08	.1	.14	− .11	− .05
**Permissive**					
*Children’s view*					
Mother	− .07	.03	− .01	.11	− .04
Father	− .11	− .1	− .14	.12	− .06
Parents	− .1	− .03	− .09	.15	− .03
*Parents’ view*					
Parents	− .09	− .01	− .291*	.05	.24
Mother	− .06	− .03	− .332*	.09	.27
Father	− .09	.01	− .1	− .02	.11
**Authoritarian**					
*Children’s view*					
Mother	.18	.09	.13	.11	− .14
Father	− .01	.13	− .03	.2	.06
Parents	.13	.15	.08	.13	− .03
*Parents’ view*					
Parents	− .03	.005	− .07	.02	− .07
Mother	− .09	− .08	− .01	− .05	− .16
Father	.07	.1	− .11	.1	.08

*p < 0.05, **p < 0.01

With respect to the emotional competence in children and parenting style, correlations revealed that only authoritative (children’s view) (*r* = .34, *p* < .05) but not authoritarian and permissive parenting style was significantly associated (see Table 7).

**Table 7:** Correlation between children’s emotional competence and parenting style

Parenting styles	Recognition of own emotions	Recognition of the emotions of others	Emotion regulation	Emotional expression	Regulation of the emotions of others
**Authoritative**					
*Children’s view*					
Mother	− .01	.3	− .01	.14	− .02
Father	.17	.31	.08	.1	.12
Parents	.1	.346*	.06	.13	.04
*Parents’ view*					
Parents	.16	− .04	.11	.27	.02
Mother	.05	.09	.11	.22	− .03
Father	.15	− .13	.02	.12	.04
**Permissive**					
*Children’s view*					
Mother	.01	.12	.09	− .29	− .005
Father	.07	− .03	.05	− .09	.11
Parents	.04	.06	.07	− .21	.09
*Parents’ view*					
Parents	.15	− .17	− .06	− .01	.12
Mother	.14	− .07	− .17	.25	.13
Father	.07	− .15	.05	− .25	.06
**Authoritarian**					
*Children’s view*					
Mother	.03	.15	.25	− .18	.03
Father	.06	.09	.02	− .21	− .14
Parents	.04	.13	.13	− .22	− .05
*Parents’ view*					
Parents	.24	− .16	− .01	.07	.14
Mother	.11	− .14	− .12	.02	− .01
Father	.28	− .03	.14	.07	.25

*p < 0.05, **p < 0.01

## Discussion

This study investigated emotional competence and its relationship to attachment styles and parenting styles in children and their parents. To do this, we analyzed the link between attachment style dimensions in children and their parents (secure, avoidant and ambivalent/anxious) and parenting style (authoritative, authoritarian and permissive) with different aspects of emotional competence (emotion expression, emotion regulation, regulation of the emotions of others, recognition of own emotions and recognition of the emotions of others). This study makes an important contribution as it seeks to bridge a deficit in research, as few studies have examined this link in both children and their parents. Further, this study examined different aspects of maternal–paternal–children’s emotional competence, which, to our knowledge, few studies have endeavored to address this phenomenon, and the focus of most other investigation was mainly on emotion regulation. Previous research has suggested that positive and negative emotions in parents affect positive and negative emotions in children (Denham & Grout, 1992; Denham, 1993). Assessing emotional competence in children and their parents in our study showed that there are significant associations between different aspects of emotional competence. Recognition of own emotions, recognition of the emotions of others, and regulation of the emotions of others have been found to be associated in parents and children. Other findings revealed a link between children’s understandings of their mothers’ and fathers’ support and better performance of self-regulation, implying that emotional support is associated with improved emotion regulation (Morris & Age, 2009; Morris, Criss, et al., 2017). Although there are few relationships between recognition of own emotions and emotion regulation within mother and father and children’s emotional competence, there are significant relations between fathers’ and mothers’ emotional expression and recognition of the emotions of others and children’s emotional competence, which is consistent with previous research that has shown that through a secure parent-child relationship, the child learns how to freely express their emotions and helps them regulate their emotions properly (Morris, Houltberg, et al., 2017). Emotional expression has been found to correlate in parents and children, suggesting that children gain an understanding of emotional expression from their parents and that they learn how to respond to positive and negative emotional expression of others (Eisenberg et al., 1992). It is worth mentioning that Wang and Saudino (2013) proposed in a twin study that emotion regulation in early childhood is affected by genetic factors. It is therefore evident that many influential aspects including hereditary factors are involved in the development of emotion regulation, and future investigation is needed to address all dimensions of emotional competence and understanding its underlying mechanisms.

### Attachment and emotional competence

The importance of the relationship with the caregiver and its impact on emotional functioning has already been proven (Cassidy & Shaver, 2018). The findings in the current study revealed several significant correlations between mothers-fathers’ emotional competence and attachment style. Maternal emotion regulation and attachment style showed significant associations, which is in line with previous results (Adam et al., 2004). Furthermore, findings demonstrated that attachment style in fathers is related to emotional competence, which has led to the hypothesis that children with secure attachment to their fathers display emotional availability in their behavior (Bowlby, 1969). Previous studies showed the increasing role of father in children’s development (Ramchandani & Psychogiou, 2009; Lamb, 2004) which is the result of fathers spending more time with their children over the past decades. It may indicate an important role of attachment of fathers which has shown that children who are securely attached to their father showed fewer behavioral problems (Verschueren & Marcoen, 1999) and proper performance in problem solving tasks (Easterbrooks & Goldberg, 1984) in comparison to insecure children. Parents’ emotional expression, regulation of the emotions of others, recognition of own emotions, recognition of the emotions of others, and emotion regulation were associated with fathers’ and mothers’ attachment style. Significant negative correlations between insecure ambivalent attachment in children and mothers’ emotional competence are consistent with previous research that emphasized a significant role of maternal attachment in children’s emotional competence (Stefanovic-Stanojevic et al., 2015). An examination of emotion regulation in mothers and secure attachment style in children indicated a significant association which is consistent with previous research on this association in middle childhood (Waters et al., 2010; Kerns et al., 2007). Hershenberg et al. (2011) also found relations between secure attachment and lower level of emotion dysregulation in parents and children.

### Parenting style and emotional competence

Another aim of the current investigation was to examine whether emotional competence in parents and their children is correlated with parenting style. Positive associations were found between authoritative style of parenting and emotional competence, which suggests that authoritative parenting might have a crucial role in the development of emotions. This is consistent with research demonstrating that authoritative parenting is related to better emotion regulation (Haslam et al., 2020; Davies & Cummings, 1994). Also, our results align with prior studies that identified negative associations between a permissive style of parenting and parents’ emotion regulation which argued that children of permissive parents have difficulties in emotion regulation (Baumrind, 1967; Jabeen et al., 2013). Results from prior studies suggested that emotion regulation in children is affected by negative parenting (Eisenberg et al., 1996; Chang et al., 2003). Furthermore, previous research has suggested that children with permissive parents are less inclined to develop emotional maturity and self-regulation (Steinberg et al., 1989; Baumrind et al., 2010). In contrast with previous findings (Shaw & Starr, 2019) which examined maternal emotion dysregulation and authoritarian parenting style, the current study did not find significant relations between authoritarian parenting and emotion regulation. This contradiction can be attributed to the differences with regard to sample and age (e.g., child sample vs adolescent sample). Future longitudinal research may decrease this sort of biases through more comprehensive examination. Another plausible interpretation of this discrepancy could be parents’ report of their own parenting style which cannot reflect children’s viewpoint (Paulson, 1994).

### Study limitations and future research

The current study consisted of a rather small sample and the findings cannot be generalized to the entire population. Future investigations on this topic should have larger samples to enable generalization of the results to different populations. Also, it should be taken into account that the use of self-report questionnaires in this study may not precisely reflect the respondents’ characteristics. Furthermore, this study was not able to examine the whole family including siblings. Therefore, future research is needed to consider all family members including siblings in order to provide a more accurate understanding of emotional competence relationship regarding to attachment and parenting style.

Despite the above limitation, this study elaborates on previous finding by including parents-children self-repot of emotional competence, attachment style, and parenting style. Furthermore, this investigation highlights the crucial role of both mothers and fathers in developing emotional competence and its relationship with attachment and parenting style. Future studies may make use of a longitudinal design to conduct more comprehensive examinations of children’s emotional competence.

## Conclusions

The findings provide initial support to the assumption that attachment is related to emotional competence in parents. Authoritative parenting and permissive parenting were significantly associated with emotional competence in parents. In addition, emotional competence in children seems to be associated with an authoritative parenting style. These results add to previous knowledge insofar as there are few studies investigating the emotional competence of parents in relation to their children’s, and they focus primarily on the mother's viewpoint. In conclusion, training parents to improve their emotional competence could strengthen the attachment security of their children.

## Data Availability

The data and materials are available on request from the corresponding author.

## Abbreviations

EKF: Emotional Competence Questionnaire
PAQ: Parenting Style Questionnaire
AAS: Adult Attachment Scale
BoBiTe: Bochumer Bindungstest
